# Modern Approaches to Lower Lipoprotein(a) Concentrations and Consequences for Cardiovascular Diseases

**DOI:** 10.3390/biomedicines9091271

**Published:** 2021-09-20

**Authors:** Victoria A. Korneva, Tatjana Yurjevna Kuznetsova, Ulrich Julius

**Affiliations:** 1 Department of Faculty Therapy, Petrozavodsk State University, Lenin Ave. 33, 185000 Petrozavodsk, Russia; eme@karelia.ru; 2 Lipidology and Lipoprotein Apheresis Center, Department of Internal Medicine III, University Hospital Carl Gustav Carus, Technische Universität Dresden, Fetscherstr. 74, 01307 Dresden, Germany; ulrich.julius@ukdd.de

**Keywords:** lipoprotein(a), proprotein convertase subtilisin/kexin type 9 inhibitors, inclisiran, lipoprotein apheresis, inhibitors of apolipoprotein(a) synthesis, cardiovascular risk

## Abstract

Lipoprotein(a) (Lp(a)) is a low density lipoprotein particle that is associated with poor cardiovascular prognosis due to pro-atherogenic, pro-thrombotic, pro-inflammatory and pro-oxidative properties. Traditional lipid-lowering therapy does not provide a sufficient Lp(a) reduction. For PCSK9 inhibitors a small reduction of Lp(a) levels could be shown, which was associated with a reduction in cardiovascular events, independently of the effect on LDL cholesterol. Another option is inclisiran, for which no outcome data are available yet. Lipoprotein apheresis acutely and in the long run decreases Lp(a) levels and effectively improves cardiovascular prognosis in high-risk patients who cannot be satisfactorily treated with drugs. New drugs inhibiting the synthesis of apolipoprotein(a) (an antisense oligonucleotide (Pelacarsen) and two siRNA drugs) are studied. Unlike LDL-cholesterol, for Lp(a) no target value has been defined up to now. This overview presents data of modern capabilities of cardiovascular risk reduction by lowering Lp(a) level.

## 1. Introduction

**Figure 1:**
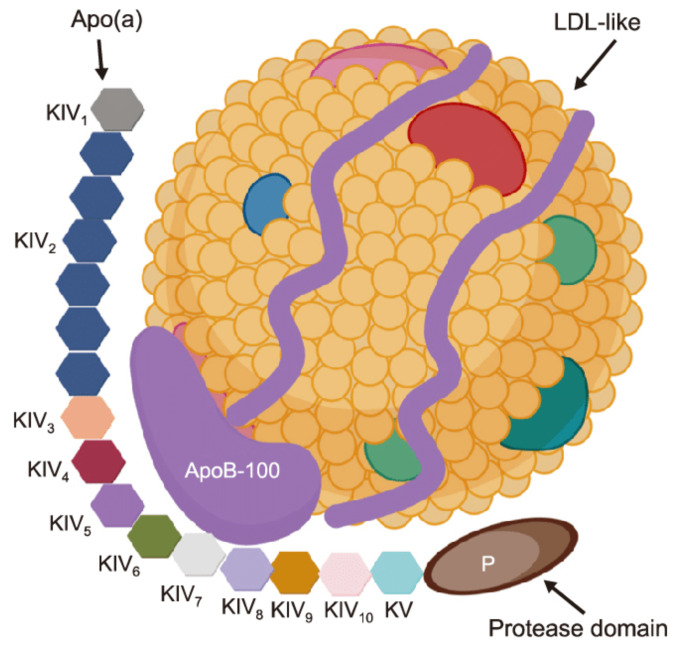
Lipoprotein(a) [Lp(a)] structure. Created with BioRender.com. Reprinted from Ref. [2].

Lipoprotein(a) (Lp(a)) is a genetically determined low density lipoprotein particle. It possesses pro-atherogenic, pro-thrombotic, pro-inflammatory and pro oxidative properties. Lp(a) contains apolipoprotein(a) and apolipoprotein B-100 moieties [1]. Structure of Lp(a) presented in Figure 1.

This lipoprotein had been detected in 1963, in the 1990s studies demonstrated its role in the development of myocardial infarction or stroke. In the last years Lp(a) was internationally recognized as an important atherogenic risk factor. Its elevation can be observed in European populations, but especially persons of African origin exhibit highly elevated Lp(a) concentrations. It has been proposed that the physiological role of Lp(a) may be to promote wound healing. Its normal concentration does not exceed 30 mg/dL (75 nmol/L), in some people no Lp(a) can be detected [3]. It is interesting that Lp(a) is only found in human beings, in old world monkeys, and hedgehogs.

Lp(a) consists of two major components. The first component is an «LDL-like» particle, its structure is similar to an LDL. This particle contains apoB-100. The second component is a specific glycoprotein apolipoprotein(a) (apo(a)), which is bound covalently to the apoB contained in the outer shell of the particle. This particle is similar to plasminogen with a size ranging from 300 to 700 kDa. [3].

LPA gene controlled Lp(a) plasma concentrations, they are individual. This gene is located on chromosome 6q26-27. The variability in size of apo(a) is caused by copy number variations within the LPA gene that determines the number of kringle IV (K-IV) Type 2 repeats [4,5]. These variable apo(a) sizes are called as “apo(a) isoforms.” A correlation was found between the apo(a) isoform size and the Lp(a) plasma concentration. The larger isoforms accumulate intracellularly and induce a slower rate of production. Lp(a) is synthesized after its release from the cells, so the larger isoforms limit the Lp(a) plasma concentration [4,5]. In contrast, a low number of isoforms is associated with a higher Lp(a) concentration.

Lp(a) influences both to the processes of atherogenesis and thrombogenesis. This lipoprotein was found to be involved in the following pathogenetic mechanisms of atherogenesis: increasing arterial wall cholesterol deposition, attracting inflammatory cells to vessel walls and inducing monocyte-chemotactic activity in sub-endothelial spaces, generation of oxidized radicals in monocytes, foam cell formation, and smooth muscle cell proliferation [1]. Lp(a) is the major carrier of a wide array of oxidized phospholipids and is the trigger of multiple proinflammatory pathways [1,6]. Lp(a) also is hypothesized to be involved in tissue repair through the interacting with components of the vascular wall.

Activation of thrombogenesis by Lp(a) is explained by the fact that the structure of apo(a) is similar to plasminogen. Lp(a) competes with plasminogen for its binding site, decreases plasmin synthesis and inhibits fibrinolysis. Lp(a) stimulates the secretion of PAI-1. However, high Lp(a) concentrations were not associated with the risk of venous thrombosis or venous thromboembolism in clinical trials [3].

A linear relationship between Lp(a) concentration and coronary heart disease incident in general population was supported by Mendelian randomization analysis [7]. Based on available data, European guidelines suggest, that an Lp(a) concentration of 50 mg/dL or higher is a potential target for treatment, and it should be mentioned, that this point is absent in the United States guidelines [8,9]. It is estimated that about 20% of a European population have elevated Lp(a) levels. Though it has to be admitted that not each person with such a feature will develop cardiovascular diseases. Evidently, additional risk factors like smoking, arterial hypertension, hyperlipidemia, renal insufficiency, and diabetes mellitus play a role. Very often patients who suffer from a myocardial infarction at a young age (before 50 years) come from families where the parents or even grandparents were affected from cardiovascular events at a comparatively young age. In the present time, new drugs are in the pipeline which will rather effectively and specifically decrease Lp(a) concentrations. Among the available drugs only PCSK9 inhibitors exert such an effect, but an elevation of Lp(a) is no officially accepted indication for their use. In some countries, first of all in Germany, high Lp(a) levels are accepted as an indication for the extracorporeal lipoprotein apheresis (LA) [8].

This review reports about the current situation with respect to the efficiency and mechanism of action of drugs decreasing Lp(a) concentrations and points out the position of LA. Special attention will be paid to outcome data for cardiovascular events.

A major problem which has not been resolved up to now is the difficulty of measuring Lp(a) concentrations. The number of Kringles IV Type 2 which is mainly responsible for the given level has an impact on the accuracy of the measurement. Previously, the mass of Lp(a) was measured (the dimension is mg/dL), more recently the number of Lp(a) particles is estimated (the dimension is nmol/L). It is not possible to convert simply one dimension into the other.

In addition, certain mutations in the Apo(a) gene may have an effect on Lp(a) concentrations, but looking for these mutations is not recommended in daily practice.

## 2. Proprotein Convertase Subtilisin/Kexin Type 9 Inhibitors (PCSK9i)

However, FDA did not approve any drugs for Lp(a) lowering. PCSK9i are one of the few pharmacotherapeutic options, which can reduce Lp(a). They can be used as monotherapy (e.g., PCSK9i), or in combination therapy (e.g., PCSK9 inhibitor + niacin) [10,11,12].

PCSK9i lower Lp(a) concentrations by approximately 25% [13,14] and it was observed, that it can reduce [15,16,17]. In the Fourier Study a weighted least square linear regression analysis that examined the association between treatment effect on CHD death, MI, or UR and per unit decrease in Lp(a) adjusting for differences in LDL-C showed a significant risk reduction for Lp(a). In the Odyssey Outcomes Study mathematical models have been used to calculate the effect of the reduction of Lp(a) by the PCSK9 inhibitor independently of the baseline and concomitant reduction of Non-HDL-C and LDL-C levels. Also, clinical and demographic variables have been taken into account. Using these models an independent effect of the reduction of Lp(a) on outcome data was shown [18,19,20]. In the FOURIER analysis the median baseline Lp(a) value was 37 nmol/L, a higher rate of major coronary events (coronary heart disease (CHD), death, MI, or urgent revascularization) in patients with higher baseline Lp(a) values was shown [19].

Greater efficacy was displayed in patients with higher baseline Lp(a), a 23% reduction with baseline Lp(a) above the median versus 7% below the median. Major coronary events were reduced by 15% per 25 nmol/L reduction of Lp(a). The ODYSSEY OUTCOMES analysis revealed a median baseline Lp(a) of 21.2 mg/dL and like the FOURIER analysis, confirmed baseline Lp(a) predicted MACE incidence. The effect of Lp(a) lowering on MACE could be shown after adjustment for demographic and clinical variables [20]. In the alirocumab group, median reduction in Lp(a) was 23.6% (5 mg/dL) and predicted reduction in MACE (HR 0.86, 95% CI 0.80–0.92), independent of LDL-C. Thus, PCSK9 inhibition may represent a therapeutic modality, that refines residual risk management beyond solely lowering LDL-C.

In practice 13% of patients have unusual responses (not optimal decreasing of LDL level) to PCSK9 inhibitors [21]. One of possible explanations for this fact is that these patients had high concentrations of Lp(a) [21]. The exact mechanism for Lp(a) clearance is not fully elucidated, but it is influenced by PCSK9 inhibitors. It was found that PCSK9i reduce LDL-C and Lp(a) in 2:1 ratio. For example, LDL-C reduced by 50–60% and Lp(a) by 25–30%. However often it may be in a discordant manner—in more than 30% Lp(a) and LDL-C do not fall concordantly [22,23]. As was shown by B. Warden, patients with unusual response to PCSK9i had a 2.5-fold higher baseline of Lp(a) compared to usual responders (73 vs. 30 mg/dL, respectively), but baseline LDL-C concentration was similar in both groups (133 vs. 132 mg/dL, respectively). Thus, the reduced LDL-C response could be accounted for the higher proportion of the LDL-C, consisting of Lp(a) particles, which are not cleared efficiently by the LDL receptor [21].

Also, a high prevalence of discordance in LDL-C and Lp(a) reduction was demonstrated in response to evolocumab, particularly when baseline Lp(a) concentrations were higher. This fact indicates the possibility of alternative pathways of Lp(a) reduction by evolocumab beyond LDL receptor (LDLR)–mediated clearance [23].

The exact mechanism of Lp(a) lowering by PCSK9i remains unknown. Current hypotheses include several assumptions: (1) increased clearance of Lp(a) particles via the LDLR [24]; (2) increased clearance of Lp(a) via additional receptors (LDL receptor-related protein 1[LRP1], cluster of differentiation 36 receptor [CD36], toll-like receptor 2 [TLR2], scavenger receptor-B1 [SR-B1], and plasminogen receptors) [25]; (3) reduction in apo(a) production, secretion, and/or assembly [26,27,28].

There are several challenges for the fact, that Lp(a) is largely cleared from the plasma via LDLR. Some of them are the following: the affinity of Lp(a) for the LDLR is far less than that of LDL [27]; Lp(a) catabolic rates are similar in familial hypercholesterolemia (FH) and non-FH patients [29]; Lp(a) levels are largely unaffected by drugs, that upregulate the LDLR (e.g., statins) [30]; PCSK9 inhibition in patients with homozygous FH (HoFH) and null LDLR mutations lowers Lp(a) more than LDL-C levels [30]; similar levels of Lp(a) were seen in carriers vs. non-carriers of loss of function PCSK9 mutation [31,32,33]; and epidemiological studies do not consistently demonstrate a correlation between plasma PCSK9 and Lp(a) concentrations [34].

If the LDLR was a major pathway for Lp(a) clearance, then PCSK9 antagonism should produce proportional reductions in LDL-C and Lp(a), with patients achieving the 2: 1 ratio (LDL-C 50–60%: Lp(a) 25–30%) seen in the large clinical trials. However, recent works has highlighted the fact that a significant proportion of patients demonstrate discordant responses with robust reductions in LDL-C but minimal or no reduction in Lp(a) [22,23]. Two recent studies have examined the issue of discordance, defined a ratio LDL-C: Lp(a) reduction as 3.5:1. This corroborates to an LDL-C reduction of ≥35% and an Lp(a) reduction ≤10% [22,23].

Patients with lower baseline Lp(a) demonstrated the greatest Lp(a) reductions in response to PCSK9 inhibition. The PROFICIO (Program to Reduce LDL-C and Cardiovascular Outcomes Following Inhibition of PCSK9 in Different Populations)—is the clinical trial program, which evaluated 895 patients receiving evolocumab [24]. Baseline LDL-C and Lp(a) concentrations were 133 and 46 mg/dL, respectively. Average reductions on evolocumab were 63.3% and 29.6%, and it confirmed the expected 2:1 ratio. The study displayed a moderate correlation (r = 0.37, *p* < 0.001) between percent LDL-C and Lp(a) reduction. Discordance was progressively more prevalent among those with higher baseline Lp(a), 10 mg/dL (19.7%), 30 mg/dL (26.5%), and >50 mg/dL (28.6%) [24].

It is conceivable that Lp(a) clearance may be dependent on apo(a) isoform size. PCSK9i induced Lp(a) reduction, which correlates to baseline Lp(a) values, with larger isoforms, signifying lower plasma concentrations, seeming to be more effectively cleared. Importantly, both of these studies suggest, that there are other mechanisms and/or pathways beyond LDLR that account for PCSK9i induced Lp(a) reductions [35].

Numerous animal studies help to interpret clinching data on the metabolism of the role of Lp(a) and its metabolic characteristics [36,37]. It was shown that modulation of LDLR expression with the PCSK9 inhibitor alirocumab did not alter the cellular or the hepatic uptake of Lp(a), LDL receptor is not a major route for Lp(a) plasma clearance [37]. This research has some limitations. The results of this work have clinical implications because they underpin why statins are not efficient at reducing Lp(a).

## 3. Other Drugs and Their Impact on Lp(a) Concentrations

### 3.1. Inclisiran

Inclisiran (Leqvio^R^), a small interfering RNA (siRNA), was shown to inhibit hepatic synthesis of the PCSK9 protein. Triantennary N-acetylgalactosamine (GalNAc) modification of the double-stranded inclisiran molecule ensures rapid hepatic uptake through the asialoglycoprotein receptors expressed exclusively on liver cells; after uptake, inclisiran is bound to the RNAinduced silencing complex in liver-cell cytoplasm. The drug is usually injected by the medical staff, the second injection takes place after 90 days, and thereafter injections are scheduled after 6 months accordingly.

Inclisiran exerts similar reductions of LDL-C concentrations like the PCSK9i (more than 50%). In the ORION 10 trial (patients with atherosclerotic cardiovascular disease), the initial median Lp(a) value was 57 nmol/L [35]. After 510 days, the reduction rate of this parameter was equal to 25.6% when comparing with the placebo group. Similar effects were seen in the ORION 11 study (patients with atherosclerotic cardiovascular disease or an atherosclerotic cardiovascular disease risk equivalent): initial median Lp(a) level was 42 nmol/L, and relative reduction was 18.6%. Thus, also when using this drug, the reduction rate of Lp(a) amounted to about half of that seen for LDL-C. In the ORION 9 investigation (in patients with heterozygous familial hypercholesterolemia) this reduction rate was less (−17.2%)—here the median Lp(a) concentration at baseline was 57 nmol/L [38]. The corresponding reduction rate of LDL-C in the latter study amounted to a between-group difference of −44.3 percentage points (taking into account the difference to the placebo group.) In none of these studies cardiovascular events were different between the verum and the placebo groups. While PCSK9i usually have to be injected in a biweekly interval (by the patients after they had been appropriately instructed). Besides local reactions at the injection sites, no adverse effects have been seen in inclisiran studies with several thousand participants.

ORION-4 (ClinicalTrials.gov identifier: NCT03705234) is a research study (planned number of patients 15,000). The main aim of this trial is to analyze the opportunity of a new cholesterol lowering injection to reduce the risk of heart attacks and strokes in patients who have already had one of these diseases or the history of surgical procedures on peripheral arteries.

### 3.2. Pelacarsen

The antisense oligonucleotide AKCEA-APO(a)-LRx (Pelacarsen) effectively reduces Lp(a) levels (up to 80%) by impairing the synthesis of apolipoprotein(a) (apo(a)) [39]. This antisense oligonucleotide is directed to hepatocytes by conjugation with a triantennary N-acetylgalactosamine (GalNAc3) moiety, a high-affinity ligand for the asialoglycoprotein receptor on the surface of hepatocytes, have resulted in large increases (by a factor of 15 to 30) in their potency, with implications for improvements in the side-effect profile and safety.

A decrease of oxidized phospholipids, associated with apo(a), was also seen (up to 41%). In the Phase II study, Pelacarsen induced only very few adverse effects, mainly reactions at the injection site. A Phase III Study, aiming at the evaluation of cardiovascular endpoints, was started in 2020 (HORIZON trial; ClinicalTrials.gov Identifier: NCT04023552). Patients who had suffered from a cardiovascular event in the last 10 years and whose Lp(a) is ≥70 mg/dL are eligible. Patients will inject 80 mg Pelacarsen once per month or the placebo. The planned duration of this study is about 4 years or until 993 cardiovascular events will occur. The concomitant LDL-C lowering therapy in these patients will be optimized. The latter therapeutic approach can also effectively reduce the incidence of new cardiovascular events. Probably, it will not be easy to show that the reduction of Lp(a) levels itself will provide an additional benefit.

### 3.3. siRNA against Apo(a)

Two companies had started studies with a small inferring RNA (siRNA) against Apo(a). Amgen has initiated a Phase 2 study enrolling 240 patients with an evidence of atherosclerotic disease and an Lp(a) >150 nmol/L in July 2020 (the name of this drug is olpasiran (AMG 890); ClinicalTrials.gov Identifier: NCT04270760). Four different doses will be tested. This study will end in 2023.

Silence Therapeutics plc began a study with another small interfering RNA against apo(a) (SLN360; ClinicalTrials.gov Identifier: NCT04606602). This trial will include 88 patients, they will be subdivided into 9 cohorts using single or multiple doses ranging from 30 to 900 mg.

If these three agents will prove to be safe and effective they will be available for the use in clinical routine in about five years.

More drugs have been seen to decrease Lp(a) concentrations: 1. Niacin—reduction by about 20%; no longer available due to no proven effect on cardiovascular endpoints in two major studies and severe adverse effects [40], 2. Estrogen—reduction by 10—15%; no longer recommended due to the observation that a hormone replacement therapy may lead to adverse events (breast cancer, thrombosis, stroke), 3. Mipomersen (antisense oligonucleotide against apolipoprotein B—reduction by (−26.4 (95% CI −42.8, −5.4), no longer recommended because adverse events occurred (reactions at the injection sites, steatosis hepatis, low efficiency of lowering of LDL cholesterol) [41], 4. Lomitapide (inhibitor of microsomal triglyceride transfer protein)—reduction by about 13%, only used in patients with homozygous familial hypercholesterolemia.

However, it remains an open question to what extent the decrease in Lp(a) with the help of medication contributes to a decrease in cardiovascular risk. Niacin reduces lipoprotein (a) by 15% to 25%, but does not reduce death or ischemic cardiovascular events [42,43]. Anacetrapib, a CETP inhibitor which is no longer available, lowers Lp(a) by 25% with only modest cardiovascular benefits, which are likely explained by other effects on the lipid profile [44].

## 4. Lipoprotein Apheresis (LA)

In the 1960s, patients with homozygous familial hypercholesterolemia were treated in Great Britain with plasma exchange. The specific LA therapy started in the 1980s in Germany and in Japan. However, the major focus was on LDL-C reduction. The significance of Lp(a) as an atherogenic risk factor only became clear after the studies published in the 1990s.

At present, an elevation of Lp(a) levels is a recognized indication for the extracorporeal therapy, which is realized in a large scale only in Germany. In 2018 among 3737 patients who were treated with lipoprotein apheresis (LA) 2136 patients (57%) were diagnosed with an isolated Lp(a) elevation (these are the latest publicly accessible data) [45].

The Joint Federal Committee (Gemeinsamer Bundesausschuss) defined this diagnosis in the following ways: (1) Lp(a) levels exceeding 60 mg/dL (or 120 nmol/L). (2) LDL cholesterol within the target levels recommended by international guidelines. This criterion cannot be reached in each patient, most probably due to the genetic background and to an intolerance of lipid-lowering drugs. Moreover, it has to be taken into consideration that a part of the measured LDL cholesterol is transported by the Lp(a) particles and is not influenced by statins. (3) A progression clinically documented or documented by imaging technique of atherosclerotic lesions (as a rule a repetitive cardiovascular event or intervention) [46].

Unfortunately, in other European countries LA does not play a major role. It is especially astonishing that the extracorporeal therapy is not performed in Denmark—several population studies come from this country. Of course, it has to be admitted that LA is a rather expensive and time-consuming therapeutic approach which needs the work of a highly qualified medical staff.

After each LA session the Lp(a) concentration is usually reduced by more than 60% (up to 90%). In fact, each available LA method is able to effectively decrease this concentration.

After several months of weekly LA sessions, the pre-session Lp(a) levels are reduced by about 25% when comparing with the concentrations measured before the start of the extracorporeal therapy [47].

In lipidology the cumulative loading of lipid concentrations plays an important role with respect to atherogenicity [47]. In this respect calculating the interval mean values (IMV) during LA therapy is a modern approach. We calculated IMV for LDL cholesterol according to Kroon [48] and for Lp(a) according to Tselmin [49].

In an evaluation published in 2020 [50], the following mean values for Lp(a) and LDL-C were seen (Table 1):

In the majority of the patients LA was performed several years ago, and the other data were observed during an LA session in 2019. The Lp(a) IMV were 50% lower than the initial concentrations, but on the other hand they are higher than optimal values. In some apheresis patients a dangerous progress of atherosclerosis was observed—it was decided to add a PCSK9 inhibitor (especially in patients with relatively high LDL-C levels) or to switch a few patients to two LA sessions per week. In the experience of the Dresden Lipoprotein Apheresis Center, in a few patients LA therapy could be stopped when the PCSK9i was started—exclusively in patients whose Lp(a) levels were not elevated. Lp(a) levels were decreased by this drug in a range from 0 to 44% (after 12 weeks). Thus, in some patients, it was possible to switch some patients to a biweekly LA regimen. Of course, patients have to be monitored on a regular basis with respect to their cardiovascular situation—if any progress is seen the weekly interval will be resumed. With respect to inclisiran, a few patients have been administered this drug, but a final opinion cannot be given yet.

Table 1 also depicts the situation with regard to LDL-C—of course all patients were on a maximal lipid-lowering therapy (statin, ezetimib, PCSK9 inhibitor) when tolerated. Pre session LDL-C were less decreased when comparing with the 1. LA than Lp(a), post session decreases were comparable, but the LDL-C IMV were relatively less decreased than the corresponding Lp(a) IMV.

When comparing two subgroups—one without cardiovascular events (CVE) during LA therapy (*n* = 60) with the other patients with CVE during extracorporeal treatment (*n* = 48)—all measured lipid concentrations (including LDL-C and Lp(a)) were not different. However, patients with CVE had started LA at a higher age and had experienced more CVE before LA initiation [50].

Unfortunately, no randomized controlled studies have been performed to prove the effectiveness of LA with respect to the reduction of CVE during extracorporeal therapy. In Germany, the authorities wanted that such a study should be undertaken, but the design was not approved by ethics committees. Thus, the only way to demonstrate the efficiency of LA in this aspect is to compare the situation before and during LA therapy. For patients with high Lp(a) levels, two studies showed a high reduction of CVE: the Jaeger study [51] and the Pro(a) Life study [52,53]. Reduction rates of up to 90% have been described, when comparing the two years anteceding LA therapy with the two years after the start of LA. This effect was seen also for the follow-up time up to five years during LA. It could be shown that the number to treat (NNT) to prevent a cardiovascular event is equal to three—this number is much smaller than that seen with lipid-lowering drugs. It was observed that an LA therapy is more effective with respect to lowering CVE rates in patients with elevated Lp(a) levels than in patients with normal or not detectable Lp(a) [47].

In a study in 20 patients with elevated Lp(a) levels and therapy-resistant angina pectoris, which included a sham apheresis, the true LA for 12 weeks improved the myocardial blood supply, reduced atherosclerotic area at the carotids, and increased physical abilities [54].

The majority of LA methods reduce both Lp(a) and LDL-C concentrations. It is not easy to differentiate these effects with regard to outcome data. However, there are Russian columns offering a specific decrease of Lp(a). In a study with 30 patients, of whom 15 were treated with this kind of LA, beneficial effects of the extracorporeal therapy (compared to statin therapy only) on coronary arteries (angiographic control after 18 months) and on the intima media thickness were demonstrated [55,56]. This study emphasizes the importance of the reduction of Lp(a) levels on atherosclerosis.

In Table 2 an overview is given on the drugs discussed in this manuscript and LA with respect to their effect on Lp(a) concentrations and published outcome data.

## 5. Conclusions

Thus, the available data from epidemiological and clinical trials clearly support the impact of increased Lp(a) concentration on cardiovascular risk. However, not all medications, which decrease Lp(a) concentration, improve the prognosis. Today, the most promising areas of therapy, that reduce both Lp(a) level and cardiovascular risk, are medications, which influence PSCK9, and LA. It is difficult to define how low Lp(a) levels should be to be relevant with respect to lowering of cardiovascular events. The officially accepted limits (60 mg/dL or 120 nmol/L) are rather high taking into account that according to observation data atherosclerosis most probably develops already at lower Lp(a) concentrations. The situation is quite different for LDL-C where clear targets have been defined.

In the literature, based on mendelian randomization study data, it was published that in order to reduce cardiovascular events by 22% (this reduction was described for the decrease of 1 mmol/L (38.67 mg/dL) of LDL-C) a decrease of 101.5 mg/dL of Lp(a) is needed [7]. In another evaluation this number was reported to be about 65.7 mg/dL [57]. The authors conclude that this estimate is determined by the observed effect estimates of single-nucleotide polymorphisms on Lp(a) concentrations and is therefore influenced by the standardization of the Lp(a) assay used. As a consequence, calculations of the required Lp(a)-lowering potential of a drug to be clinically effective might have been overestimated in the past. Unfortunately, both numbers are not reflecting the real-world situation. The divergent results from clinical trials may be linked to renal effects of Lp(a) that interfere with atherosclerosis in terms of hard endpoints such as cardiovascular outcome. Although PCSK9 is expressed mainly in the liver, it was shown it expressed in other tissues and organs with specific functions (in the vascular wall, in the kidneys, and in the brain) [58].

New drugs inhibiting the synthesis of apolipoprotein(a) which are evaluated in studies nowadays will effectively reduce Lp(a) concentrations, much lower Lp(a) concentrations are seen with these drugs. However, their effects on cardiovascular morbidity and mortality will still have to be demonstrated. In particular, new studies with long-term observation are needed. At present, the only officially accepted indication to decrease the cardiovascular risk is by reducing Lp(a) concentrations existing for LA—which is broadly used only in a few countries.

## Figures and Tables

**Table 1:** Comparison of Lp(a) and LDL-C concentrations measured before (pre), after (post) and IMV at an LA session in 2019 with the initial Lp(a) and LDL-C levels before the 1st LA session.

Parameter	Lp(a) nmol/L	% in Comparison with 1. LA	LDL-C mmol/L	% in Comparison with 1. LA
1st LA session	243.5	baseline	2.7	baseline
pre session	177	−27.3	2.3	−12.8
post session	43.5	−82.1	0.7	−75
IMV	121.6	−50	1.9	−29.5

Dark pink—Lp(a) concentration, blue—LDL-C concentration, green—regime of therapy.

**Table 2:** Drugs and Lipoprotein apheresis (LA)—effects on Lp(a) levels, published outcome data, accepted indication for lowering Lp(a), current availability.

Drugs/LA	Effect on Lp(a) Level	Published EndPoint Data	Accepted Indication for Lowering of Lp(a)	Availability at Present
Statins	No effect or slight increase	An elevated Lp(a) was a relevant atherogenic risk factor in statin studies	No	Yes
PCSK9i	Minus 25–30%	An additional benefit exceeding that explained by the decrease of LDL-C was shown	No	Yes
Niacin	Minus 20%	Yes (no effect seen)	No	Yes (in USA)
Inclisiran	Minus 20–26%	No	No	Yes
Pelacarsen	Minus up to–80%	No	Yes (in the future)	No
siRNA	Not known	No	Yes (in the future)	No
Mipomersen	Minus 26.4%	No	No	No
Lomitapide	Minus 3%	No	No	In some countries
Estrogens	Minus 10–15%	No	No	Yes
Anacetrapib	Minus 25%	Yes (but unclear what was the effect mediated by the reduction of Lp(a))	No	No
